# A Novel Moderately Thermophilic Facultative Methylotroph within the Class *Alphaproteobacteria*

**DOI:** 10.3390/microorganisms9030477

**Published:** 2021-02-25

**Authors:** Tajul Islam, Marcela Hernández, Amare Gessesse, J. Colin Murrell, Lise Øvreås

**Affiliations:** 1 Department of Biological Sciences, University of Bergen, Thormøhlensgate 53 B, P.O. Box 7803, 5006 Bergen, Norway; tajisl@hotmail.no; 2 Bergen Katedralskole, Kong Oscars Gate 36, 5017 Bergen, Norway; 3 School of Environmental Sciences, University of East Anglia, Norwich Research Park, Norwich NR4 7TJ, UK; marcela.hernandez@uea.ac.uk (M.H.); j.c.murrell@uea.ac.uk (J.C.M.); 4 Institute of Biotechnology, Addis Ababa University, Addis Ababa P.O. Box 1176, Ethiopia; amare.gessesse@gmail.com; 5 Department of Biological Sciences and Biotechnology, Botswana International University of Science and Technology, Private Bag 16, Palapye 10071, Botswana

**Keywords:** thermal spring, methanol oxidation, moderately thermophilic, facultative, Alphaproteobacteria

## Abstract

Methylotrophic bacteria (non-methanotrophic methanol oxidizers) consuming reduced carbon compounds containing no carbon–carbon bonds as their sole carbon and energy source have been found in a great variety of environments. Here, we report a unique moderately thermophilic methanol-oxidising bacterium (strain LS7-MT) that grows optimally at 55 °C (with a growth range spanning 30 to 60 °C). The pure isolate was recovered from a methane-utilizing mixed culture enrichment from an alkaline thermal spring in the Ethiopia Rift Valley, and utilized methanol, methylamine, glucose and a variety of multi-carbon compounds. Phylogenetic analysis of the 16S rRNA gene sequences demonstrated that strain LS7-MT represented a new facultatively methylotrophic bacterium within the order *Hyphomicrobiales* of the class *Alphaproteobacteria*. This new strain showed 94 to 96% 16S rRNA gene identity to the two methylotroph genera, *Methyloceanibacter* and *Methyloligella.* Analysis of the draft genome of strain LS7-MT revealed genes for methanol dehydrogenase, essential for methanol oxidation. Functional and comparative genomics of this new isolate revealed genomic and physiological divergence from extant methylotrophs. Strain LS7-MT contained a complete *mxaF* gene cluster and *xoxF1* encoding the lanthanide-dependent methanol dehydrogenase (XoxF). This is the first report of methanol oxidation at 55 °C by a moderately thermophilic bacterium within the class *Alphaproteobacteria*. These findings expand our knowledge of methylotrophy by the phylum *Proteobacteria* in thermal ecosystems and their contribution to global carbon and nitrogen cycles.

## 1. Introduction

In geothermal habitats, methane and other natural gases (such as short-chain alkanes) enter the Earth’s atmosphere through gas venting, seeps, and degassing of spring water. In the Ethiopian Rift Valley region, hot spring sediments from thermal springs may promote microbial community structure and diversity. Thermal environments are suitable habitats for moderately thermophilic methylotrophs, and they may play an important role in the global methane cycle [1,2]. Biological oxidation of methanol to CO_2_ by methylotrophs in terrestrial environments reduces methanol emissions to the atmosphere and has an important effect on methanol concentrations in the atmosphere [3]. Aerobic methane- and methanol-oxidising bacteria are a unique set of Gram-negative bacteria that use reduced carbon compounds containing no carbon–carbon bonds (methane, methanol, methylated amines, etc.) as their sole carbon and energy source and make a considerable contribution to the biogeochemical cycling of C1 compounds in different ecosystems [4]. Most of the methanol-oxidizing bacteria are defined as facultative or strictly aerobic, and taxonomical studies of these bacteria have defined several different phyla of *Proteobacteria, Verrucomicrobia, Cytophagales, Bacteriodetes, Firmicutes* and *Actinobacteria* that grow on methanol. Several of the non-methanotrophic methanol oxidizers have a facultative methylotrophic lifestyle, although some species are limited to C_1_ substrates (for example *Methylophilus, Methylovorus, Methylophaga* and *Methylobacillus*) [3,5].

In the process whereby methanol is oxidized to formaldehyde, two distinct methanol dehydrogenase (MDH) enzymes are known; a calcium-dependent MxaFI type and a lanthanide-containing XoxF type MDH. Especially in methylotrophic *Proteobacteria*, the key MDH enzyme (a pyrroloquinoline quinone dependent methanol dehydrogenase enzymes) is present. The genes encoding the MDH subunits (*mxaFI*) and the cytochrome c electron acceptor (*mxaG*) were initially studied in *Methylobacterium extorquens* (reviewed in [5,6]). Functional molecular marker genes such as *mxaF* (encoding the large alpha-subunit of MDH), are highly conserved among the methylotrophs and have been used as functional genes in environmental studies to identify methylotrophs in numerous habitats [7,8]. *xoxF* encodes the alternative MDH, and has also been found in many methylotrophs [9,10] and can also be used as a functional gene marker in environmental studies [11,12].

The family *Hyphomicrobiaceae* belongs to the order *Rhizobiales* (*Hyphomicrobiales*) of the class *Alphaproteobacteria*, which is a morphologically, metabolically, and ecologically diverse group [13]. A total of 28 genera of this family have validly been described (www.bacterio.net/-classifgenerafamilies.html # *Hyphomicrobiaceae*, (accessed on 9 May 2020)). Most of these genera are aerobic chemoheterotrophs and facultatively methylotrophs, whereas some can grow anaerobically by denitrification or fermentation. Research on *Hyphomicrobiaceae* has mainly focused on low to moderate temperature ecosystems, and the majority of these described species are found to be mesophilic and neutrophilic, and have been successfully recovered from marine and non-marine habitats, including saline environments [14,15].

The species *Dichotomicrobium thermohalobium* within the family *Hyphomicrobiaceae*, was defined as a chemoorganotroph and moderately thermophile (growth temperatures between 35 and 55 °C). This budding bacterium with dichotomously branching hyphae is halotolerant, and several *Dichotomicrobium*-like bacteria have been isolated from the hypersaline Solar Lake [16]. However, at a lower temperature (4.5 °C), similar bacterial morphotypes have also been observed in Lake Constance at between 10 and 150 m depth. However, cultivation of these bacteria was not successful [17].

A limited number of facultative methanol-oxidizers within the family *Hyphomicrobiaceae* of the order *Rhizobiales* has been described. Only one moderately thermophilic species, *Methyloceanibacter caenitepidi*, with a growth temperature of 19 to 43 °C (optimum 35 °C), has been reported, and this is a facultative methylotroph, which was isolated from marine sediments near a hydrothermal vent [18]. In 2013, two non-pigmented moderately halophilic bacteria—*Methyloligella halotolerans* and *Methyloligella solikamskensis*—were isolated from saline environments (temperature range: 10 to 40 °C). Both isolates can grow on methanol and were designated obligately methylotrophic [19]. All species within the genus *Hyphomicrobium* are metabolically flexible, and grow on methanol, methylamine, di- and trimethylamine, dichloromethane and methylsulfate as their carbon and energy source, as well as some organic compounds. They are facultatively methylotrophs and are mostly distributed in soils and aquatic habitats [14,20]. Most species within the *Alphaproteobacteria* have distinct metabolic properties (chemolithoautotrophic, strictly organotrophic or facultatively chemolithoorganotrophic, photoautotrophic and some contain bacteriochlorophyll), and exhibit mesophilic growth below 45 °C. A small number of validly described moderately thermophilic genera have been isolated from hot springs. These include *Rubellimicrobium thermophilum* (45 to 54 °C), *Chelatococcus sambhunathii* (37 to 42 °C), *Tepidamorphus gemmatus* (45 to 50 °C), *Elioraea thermophila* (55 °C) and *Porphyrobacter tepidarius* (40 to 48 °C) [21,22,23,24,25]. However, none of these bacteria were reported to be obligate or facultative methanol oxidizers (presumably because they lack the enzymes for the oxidation of methanol to formaldehyde).

The isolation of moderately thermophilic and facultative methanol-oxidizers (optimally growth >50 °C) from extreme environments is challenging. In methane enrichments, methane oxidizers can produce methanol or other substrates (such as acetate), and thus cross-feeding can occur, stimulating methanol oxidizers and other non-methylotophic microorganisms to grow. It can be time-consuming to cultivate a pure culture of methanol oxidizers and also to separate these from potential methane oxidizers. An obligately methylotrophic bacterium (growing on methane and methanol at optimal temperatures of 50 to 55 °C) in the family *Methylococcaceae* of the class *Gammaproteobacteria* was isolated from an alkaline thermal spring in the Ethiopian Rift Valley and recently described [2]. Here, we present the isolation, characterization, physiology and genomic features of a moderately thermophilic and facultatively methylotrophic bacterium, which was recovered from a co-culture with a gammaproteobacterial methanotroph recovered from an alkaline thermal spring sample in the Ethiopian Rift Valley. The strain probably represents a new methylotrophic genus within the *Alphaproteobacteria*.

## 2. Materials and Methods

### 2.1. Sampling Procedures and Cultivation Strategy

Samples were harvested in November 2007 from a hydrothermal spring site within the Ethiopian Rift Valley. Water–sediment samples were collected from one of the largest hydrothermal ponds in the area showing diffuse venting and emitting high-temperature fluids. The sampling site was chosen at a location (7°28′ 666″ N and 38°38′086″ E) and had a temperature of 55.4 °C and pH 8.8. Samples (a mixture of water and sediments) were collected in a sterile beaker and then immediately transferred to Falcon tubes and stored at 4 °C in the dark until cultivation. For the initial methanotrophic enrichment culture (cultivation of moderately thermophilic methanotrophs), 3 mL sediment slurry was added to 15 mL low-salt mineral medium (LMM) supplement with KNO_3_ in 120 mL serum flasks [26]. The final pH of the medium was adjusted to 7.0. In addition, a low-salt mineral medium (supplemented by either NH_4_Cl (LMM-AC) or (NH_4_)_2_SO_4_ (LMM-AS)) was utilized for methanotrophic enrichment cultures. The following vitamin solutions were filter sterilized and added to growth medium: (1) 100 μL of a vitamin stock solution containing (mg·L^−1^) thiamine hydrochloride, 10; biotin, 1; nicotinic acid, 20; pyridoxamine, 10; cyanocobalamin (vitamin B12), 5; p-aminobenzoic acid, 10; riboflavin, 20; and (2) 1 mL of a phosphate buffer stock solution containing (g·L^−1^) KH_2_PO_4_, 37.5, Na_2_HPO_4_·2H_2_O, 49; and (3) 100 μL of a selenite-tungstate solution containing (mg·L^−1^) NaOH, 400, Na_2_SeO_3_·5H_2_O, 6, Na_2_WO_4_·2H_2_O, 8. Further enrichment procedures were as described previously [2,26].

### 2.2. Isolation and Purification of Strain LS7-MT

Multiple dilution series were prepared using the same mineral medium, and sequential transfers were performed at one-week intervals. Finally, serial dilutions in LMM were made and spread onto Gelrite plates containing LMM. Plates were incubated for four weeks at 55 °C in jars filled with a methane and air mixture (4:1). After plating on gelrite plates, we observed shiny white colonies. The colonies were transferred to fresh liquid LMM with methane and a low concentration of methanol [15 mL LMM + 0.1 µL methanol (3.12 µM)]. Growth at 55 °C was observed only with a low concentration of methanol. No growth was found with just methane. Several small, non-pigmented white colonies were recovered from Gelrite plates, examined for purity by phase-contrast microscopy and then transferred to fresh liquid LMM containing methanol (0.1% *v*/*v*) and incubated at 55 °C. Cells were harvested for subsequent analysis at the early stationary phase (between five and seven days). After isolation of the methanol-grown strain, designated LS7-MT, from Gelrite plates, cells were purified and verified by the following analyses: phase-contrast (Nikon, Eclipse E400 microscope) and electron microscopy (Jeol-1230), and re-streaking onto R2A agar plates (Difco). Utilization of methanol at concentrations from 0.05 to 2% (*v*/*v*) was determined in LMM supplemented with methanol. For measuring the range of temperatures and optimum growth conditions, cultures were tested in flasks containing LMM and 0.15% methanol at nine temperatures ranging from 25 to 65 °C. The pH (9 values from 5.0 to 10.0) and salt-dependence (NaCl) of growth (0.1, 0.5, 1.0, 2.0, and 3.0% *v*/*v*) were examined at the optimum growth temperature (55 °C). The optical density of the cultures was measured at 600 nm using a spectrophotometer. Subsequently, methanol (0.15%; *v*/*v*) was routinely used as a carbon source for the purified strain LS7-MT at 55 °C. The doubling time and specific growth rate (at 55 °C and pH 7) on methanol were calculated from the exponential growth phase of strain LS7-MT. Nitrogen sources for growth were tested by replacing KNO_3_ in LMM with 0.1 g L^−1^ of NH_4_Cl, (NH_4_)_2_SO_4_, NaNO_2_, glycine, methylamine, trimethylamine or yeast extract. The growth of strain LS7-MT was tested with nitrogen-free LMM (without KNO_3_) in triplicate serum bottles where the only available nitrogen source was the dinitrogen from the air (20% air in the headspace). To eliminate possible trace nitrate carry-over from the original inoculum, and to verify that the strain was able to grow without KNO^3−^ in the medium (No traces of KNO^3−^ in the LMM) the cultures were transferred three times. Cultures were incubated for three weeks. The growth of strain LS7-MT on a variety of organic compounds including acetate (18 mM), pyruvate (10 mM), lactate (10 mM), glucose (10 mM), maltose (10 mM), lactose (10 mM), and formate (0.03%) was investigated. These were added (filter-sterilised) to LMM containing vitamins [26]. The antibiotic sensitivity of strain LS7-MT was performed using the following antibiotics: ampicillin 10 µg mL^−1^, kanamycin 30 µg mL^−1^, streptomycin 10 µg mL^−1^, erythromycin 10 µg mL^−1^, tetracycline 10 μg mL^−1^ and nalidixic acid 30 µg mL^−1^ as described previously [26]. Growth was examined after incubation for 10 days.

### 2.3. Morphology, Electron Microscopy and Cellular Fatty Acid Analysis

The morphology of strain LS7-MT was determined using phase-contrast microscope and electron microscopy. For the preparation of ultrathin sections, cells of the exponentially growing cultures were collected by centrifugation and fixation using a protocol described previously [27]. For fatty acid analysis, cultures of strain LS7-MT grown at optimum temperature were shipped to DSMZ where the samples were processed further (by harvesting, saponification, methylation, extraction and base wash prior to GC analysis). The fatty acid patterns were compared with the patterns stored in the fatty acid database of the Microbial Identification System (MIS).

### 2.4. Genome Analysis

Total genomic DNA was extracted from strain LS7-MT, and the genome was sequenced at the Norwegian Sequencing Center (Oslo, Norway) using a 454 GS FLX sequencer (Roche, Basel, Switzerland). The genome of strain LS7-MT was integrated into the MicroScope annotation platform [28]. A phylogenomic tree based on the whole-genome sequencing analysis were created using the automated multi-locus species tree (autoMLST) pipeline [29]. AutoMLST determines closely related genomes based on alignment of >70 core genes, and the closest species were determined based on percent average nucleotide identity (ANI). Amino-acid comparisons between strain LS7-MT and their closest relative strains were calculated based on reciprocal best hits (two-way amino acid identity AAI) using the enveomics collection (http://enve-omics.gatech.edu/ (accessed on 17 February 2021)). Digital DNA–DNA hybridization (dDDH) was conducted using the Type Strain Genome Server (TYGS) [30]. Automatic annotations were validated manually for the genes involved in metabolic pathways of interest, such as methanol and formaldehyde oxidations. Evolutionary analyses of key methylotrophic genes/enzymes were conducted in MEGA X and trees (16S rRNA genes and MxaF/XoxF1) were drawn using the maximum-likelihood method, which is performed in MEGA X [31,32]. The genome sequence of strain LS7-MT has been deposited in the JGI IMG/ER database under accession 2517572012.

## 3. Results and Discussion

### 3.1. Isolation of a Moderately Thermophilic Methylotroph

Following two weeks of incubation at 55 °C with methane as the only carbon source, the microbial growth on KNO_3_ (LMM) was observed and further confirmed by phase-contrast microscopy. After three weeks of incubation under the same conditions, no growth was found on either NH_4_Cl (LMM-AC) or (NH_4_)_2_SO_4_ (LMM-AS) media. Two different types of colonies appeared on the Gelrite plates. One type consisted of small white colonies about 0.6 to 0.8 mm in diameter (comprised of coccoid cells) and the other very shiny white colonies about 1.2 to 1.5 mm in diameter (comprised of small rod-shaped cells). Five colonies of each type were selected and transferred to fresh liquid LMM (KNO_3_) with methane as the only carbon source. We observed that all five small white colonies sustained grown with methane, whereas the other five shiny white colonies did not show any indication of growth with methane even after four weeks of incubation. The small white colonies, containing the coccoidal cells growing on methane were further characterized and a description of these methanotrophs is now published [2]. The shiny white colonies that did not grow on methane were subjected to further investigation, described here. We tested the potential of these isolates to grow with methanol as their sole carbon and energy source and (as a control) for growth on R2A agar plates. The isolate recovered from a co-culture with a methanotrophic bacterium (LS7-MC) [2] was termed strain LS7-MT, and could use methanol, but not methane, for growth. Strain LS7-MT could also grow on acetate, pyruvate, glucose, methylamine, trimethylamine and R2A agar plates, indicating that strain LS7-MT is an aerobic facultative methylotroph. Strain LS7-MT also produced shiny white colonies on Gelrite plates that had no added carbon, which indicated that this strain was able to scavenge trace carbon from the medium. Heterotrophic contaminants were not observed during re-streaking on R2A agar plates, corroborating the purity of the isolate. Methanol, glucose and R2A agar plates were routinely inoculated and incubated at 55 °C for further purity tests.

### 3.2. Physiological Properties of Strain LS7-MT

The fastest growth rate of strain LS7-MT occurred between 50 and 55 °C with an initial pH of 7.0. The temperature range for growth was between 30 and 60 °C, and no growth was observed at 25 or 62 °C. The pH range for growth was 6.0 to 9.3 (optimum 7.0 and 7.5); it did not grow at pH 5.0 or 9.5. Growth did not occur under aerobic conditions in the absence of methanol or under anaerobic conditions in the presence of methanol and nitrate, indicating that this strain could probably not denitrify. Strain LS7-MT was capable of growth at up to 0.5% (*v*/*v*) methanol. When comparing nitrogen sources for growth, using growth on LMM (KNO_3_) with methanol or glucose as controls, no growth of LS7-MT was observed in media containing NH_4_Cl (LMM-AC) or (NH_4_)_2_SO_4_ (LMM-AS). This indicates that nitrate is an essential inorganic nitrogen source for growth. The same observation was also seen in the gammaproteobacterial methanotrophic strain LS7-MC [2]. Growth in nitrogen-free LMM (without KNO_3_) was not observed.

Strain LS7-MT was tested for methylotrophic and heterotrophic growth on a range of C_1_ compounds, organic acids and sugars. Strain LS7-MT grew on all multicarbon substrates tested (Table 1). No growth was observed without vitamins, indicating that vitamins in LMM are necessary for growth. Strain LS7-MT did not require additional NaCl for growth in LMM but was able to grow on a medium containing a NaCl concentration up to 0.5% (*w*/*v*). Therefore, strain LS7-MT is not a halophilic methylotroph. The doubling time (g) and specific growth rate (μ) of strain LS7-MT at 55 °C in LMM containing 0.15% (*v*/*v*) methanol were 15 h and 0.046 h^−1^, respectively. Growth was inhibited by all the tested antibiotics. Major characteristics of the facultatively methylotrophic strain LS7-MT and comparisons with other related alphaproteobacterial strains (thermophiles, moderately thermophiles and mesophiles) are provided in Table 1.

### 3.3. Identification of Phospholipid Fatty Acids (PLFA)

PLFA profiles can be used as biomarkers for active methylotrophic bacteria in situ [34]. The PLFA composition for strain LS7-MT showed a unique fatty acid profile compared with other related alphaproteobacterial species (Table 2). The major components of the PLFA compliment of strain LS7-MT were C18:0 (40.45%), C18:1w7c (24.20%) and C19:0 *cyc* w8c (20.77%). A high level of C18:1w7c fatty acid is a very common feature within the alphaproteobacterial methylotrophs. The content of C18:0 fatty acid was significantly higher in strain LS7-MT than any other methylotrophs reported [18]; however, it is important to point out that the fatty acid composition can change with different growth conditions and that the data with other methylotrophs could also change with different growth conditions.

### 3.4. Microscopic Observations

**Figure 1:**
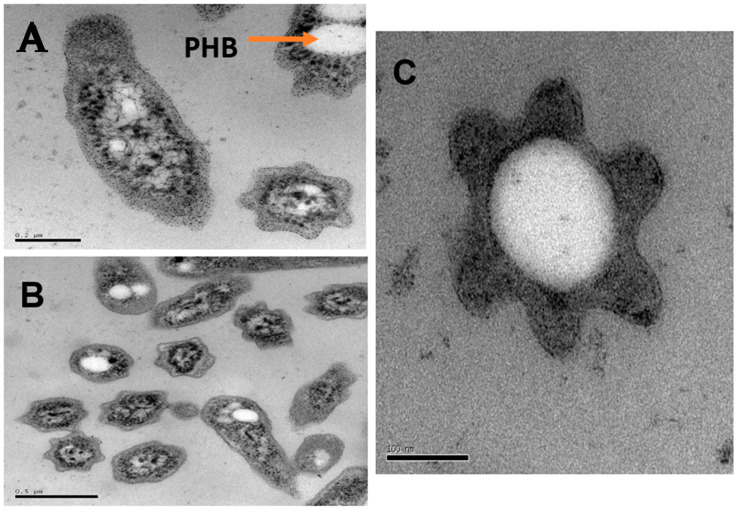
Photomicrographs of the facultatively thermophilic methylotroph strain LS7-MT. Transmission electron micrographs (TEM) of a thin section of LS7-MT cells were grown with methanol. Bars, 0.2 µm (**A**), 0.5 µm (**B**) and 100 nm (**C**). PHB, poly-hydroxybutyrate.

Only non-motile, rod-shaped cells were observed using phase-contrast microscopy. In a methanol-grown culture, the cells of strain LS7-MT occurred individually or in aggregates with a length of 0.5 to 1.5 µm and a diameter of 0.2 to 0.5 µm (Figure 1). Cells became elongated (0.4 to 1.5 µm) when growing on glucose as the only carbon source. Flagella were not apparent by electron microscopy. Intracytoplasmic membrane (ICM), carboxysome-like structures or vesicular membranes were also absent, which is consistent with strain LS7-MT being non-methanotrophic. Electron microscopy of ultrathin sections of strain LS7-MT cells revealed a typical Gram-negative cell wall structure and large poly-β-hydroxybutyrate granules (PHB). Hyphae, prosthecae or dichotomously branching hyphae (i.e., monopolar or bipolar filamentous which are common in some genera of the family *Hyphomicrobiaceae*) were not observed during growth on methanol, glucose and R2A agar plates.

### 3.5. Phylogeny of Strain LS7-MT

**Figure 2:**
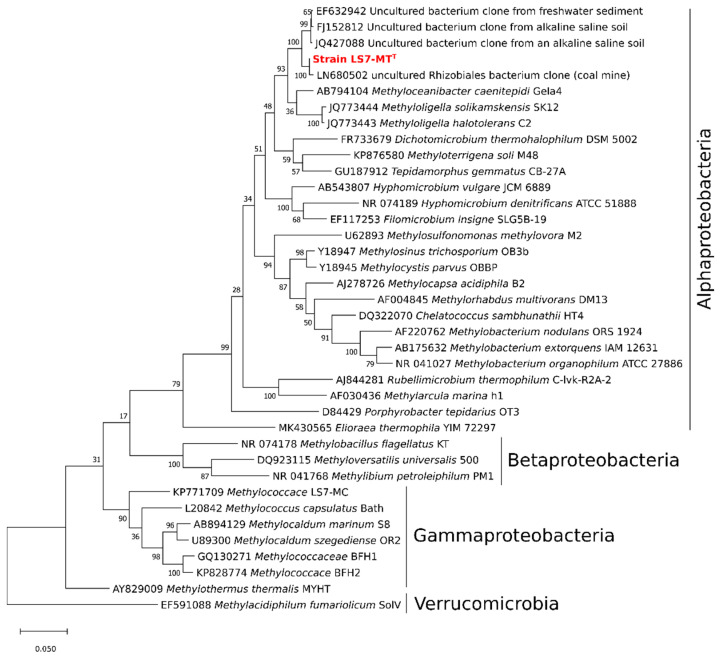
16S rRNA gene phylogeny showing the relationship between strain LS7-MT (indicated in red) and other described methylotrophs of the class *Alphaproteobacteria*, and related environmental clones. The tree is based on the maximum-likelihood method with a JTT matrix-based model [36]. The tree was built with 38 nucleotide sequences. Bootstrap values (100 replications) are shown at the nodes. The evolutionary analysis was conducted in MEGA X [31,32]. Methylotrophs of the *Gammaproteobacteria* and *Betaproteobacteria* were used as an outgroup. Genbank accession numbers are given in front of the respective strain names. Bootstrap values (100 replications) are shown next to the branches.

Analysis of the 16S rRNA gene sequence of strain LS7-MT using BLAST showed that the most closely related cultivated strains were *Methyloceanibacter caenitepidi* Gela4^T^ (96.3% sequence identity), which is a facultative methylotrophic bacterium isolated from a methane-utilizing mixed culture of marine sediment near a hydrothermal vent [18]. The sequence also showed 94.5 and 94.1% sequences similarity to *M. halotolerans* C2^T^ and *M. solikamskensis* SK12^T^, respectively [19]. Both of these strains are non-pigmented halotolerant obligately methylotrophic bacteria and were isolated from saline environments [19]. Moreover, low sequence identities (86 to 91.1%) were found to facultative methylotrophic bacteria such as *Methylobacterium nodulans, Methylobacterium organophilum, Methylobacterium extorquens, Hyphomicrobium denitrificans, Hyphomicrobium methylovorum,* and *Hyphomicrobium vulgare*. These findings suggest that the facultative methylotroph strain LS7-MT is most probably a new genus within the *Alphaproteobacteria*. The phylogenetic tree of the 16S rRNA gene indicates that the strain LS7-MT clusters together with uncultured bacteria from the order *Rhizobiales* (*Hyphomicrobiales*) of the class *Alphaproteobacteria* (Figure 2).

**Figure 3:**
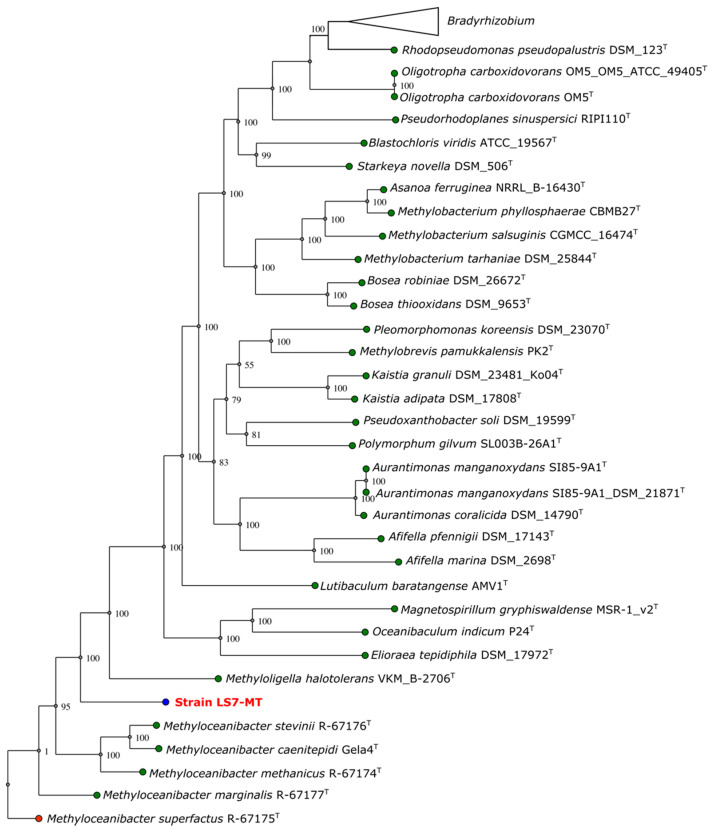
Multi-locus phylogenetic tree of strain LS7-MT using default parameters (>70 core genes) by autoMLST. Bootstrap confidence levels (1000 replicates) are indicated at internodes. Green: type strains; blue: strain LS7-MT, red: outgroup.

The 16S rRNA gene tree analysis was also supported by the whole-genome sequencing analysis using the automated multi-locus species tree (autoMLST) pipeline. The estimated average nucleotide identity (ANI) values of strain LS7-MT with genomes of different closely related strains were 76.08% with *Methyloceanibacter stevinii* and 75.93% with *Methyloceanibacter caenitepidi* GEla4^T^. The average amino acid identity (AAI) values of strain LS7-MT were 67.76% with *Methyloceanibacter stevinii* and 68.51% with *Methyloceanibacter caenitepidi* GEla4^T^. Pairwise comparisons using TYGS showed dDDH sequence identities of 20.2% to *Methyloceanibacter marginalis* R-67177^T^, 19.8% to *Methyloceanibacter superfactus* R-67175^T^ and 19.4% with *Methyloceanibacter caenitepidi* Gela4^T^. Strain LS7-MT clusters separated from these closest relatives, which indicates that it belongs to a new genus (Figure 3).

**Figure 4:**
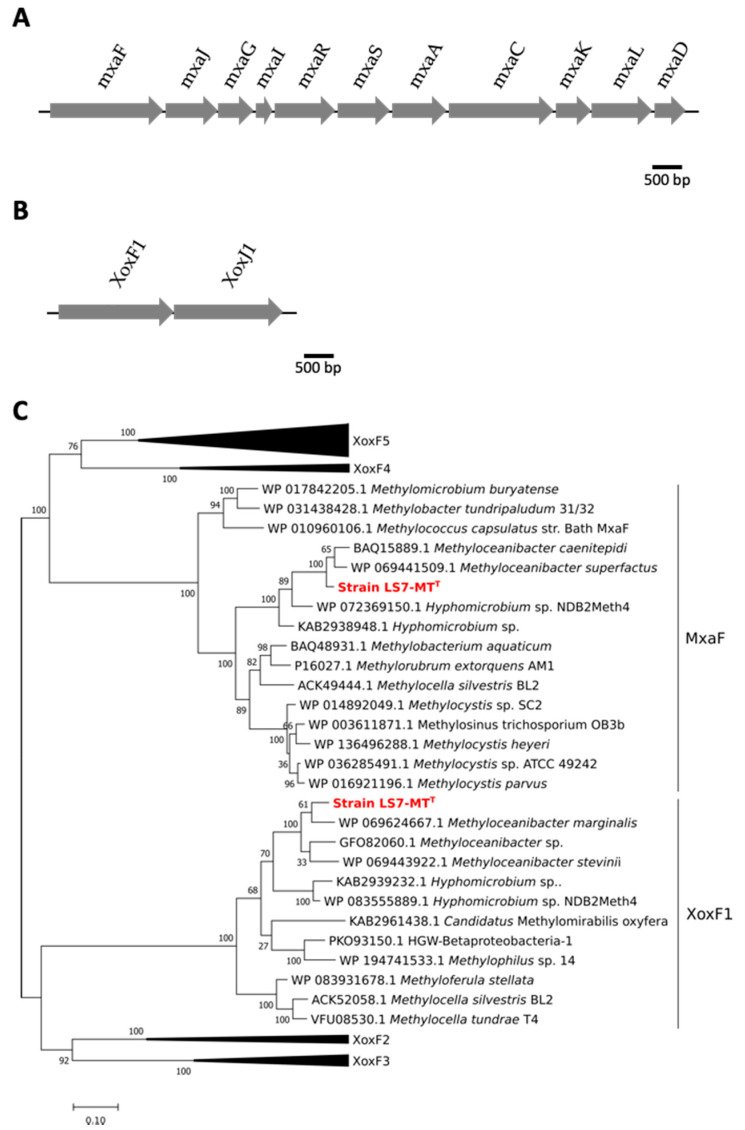
Methanol dehydrogenase gene clusters in strain LS7-MT: (**A**) the *mxaF* gene cluster; (**B**) the *xoxF* gene cluster; (**C**) phylogenetic tree of the methanol dehydrogenases MxaF and XoxF1 using the maximum-likelihood method with a JTT matrix-based model [34]. The tree was built with 65 amino acid sequences. Bootstrap values (100 replications) are shown at the nodes. The evolutionary analysis was conducted in MEGA X [31,32].

A comparison of the major characteristics of the strain LS7-MT and other genera of moderately thermophilic methylotrophs is shown in Table 1. Genes encoding the *mxa* gene cluster essential for methanol oxidation using the PQQ-dependent methanol dehydrogenase were clustered on the genome of strain LS7-MT (Figure 4A). Genes encoding *xoxF1* and its cognate gene *xoxJ1* were also found together in the genome of strain LS7-MT (Figure 4B). Phylogenetic analyses of *mxaF* and *xoxF1* gene sequences from strain LS7-MT revealed that these genes were most closely related to *mxaF* from *Methyloceanibacter superfactus* and *Methyloceanibacter caenitepidi* and to *xoxF1* from *Methyloceanobacter marginalis*, respectively (Figure 4C).

### 3.6. Genome Assembly and Annotation

To obtain a better understanding of methanol metabolism in strain LS7-MT, we sequenced and assembled the draft genome. The draft genome of strain LS7-MT comprised a single circular molecule of 2,954,375 bp sequence consisting of 194 contigs, with a total number of genes of 3152 and 3098 protein-coding genes. The genome is 97.16% complete and 1.11% contaminated. The mol% G + C content of DNA was 65.9%. Genome features are summarized in Table 3.

### 3.7. Methanol Metabolism

Genes encoding all the steps of methanol oxidation to carbon dioxide were detected in the draft genome of the isolate. Genes encoding sMMO (soluble methane monooxygenase) and pMMO (particulate methane monooxygenase) were absent from the genome of the strain LS7-MT. In methylotrophs, there are generally two types of methanol dehydrogenases. The MxaF is a calcium-dependent, pyrroloquinoline quinone (PQQ)-linked heterotetrameric enzyme, whereas XoxF is a lanthanide-dependent homodimer. A single gene cluster (*mxaFJGIRSACKLD*) encoding the large subunit and small subunit of MDH, MxaF and MxaI and accessory genes were clustered on the genome (Figure 4A). The lanthanide-dependent methanol dehydrogenase XoxF1 was also present in the genome of LS7-MT (Figure 4B). No gene encoding the enzyme MDH2 (an alternative type of MDH) was found in the genome of LS7-MT.

Screening the genome of LS7-MT revealed the presence of genes encoding enzymes of the tetrahydromethanopterin (H_4_MPT) and tetrahydrofolate (H_4_F) pathways for formaldehyde oxidation. These are crucial steps in methylotrophic metabolism since formaldehyde is a cytotoxic compound. For the tetrahydromethanopterin (H_4_MPT) pathway, formaldehyde-activating enzyme (*fae*), methenyl-H_4_MPT cyclohydrolase (*mch*), NAD(P)-dependent methylenetetrahydromethanopterin dehydrogenase (*mtdB*) and a formylmethanofuran-tetrahydromethanopterin formyltransferase (*ffsA*) were present. For the tetrahydrofolate (H_4_F) pathway, genes encoding enzymes involved in two pathways, including the methylene-H_4_F dehydrogenase and the methenyl-H_4_F cyclohydrolase enzymes were present. The genome of strain LS7-MT contains genes encoding the bifunctional enzyme FolD (5,10-methylene-tetrahydrofolate dehydrogenase and 5,10-methylene-tetrahydrofolate cyclohydrolase) encoding methylene-H_4_F dehydrogenase and methylene-H_4_F cyclohydrolase activities. The genome of strain LS7-MT also contains *fhs*, encoding formate-tetrahydrofolate ligase enzyme, *metF* encoding the methyl-H_4_F reductase enzyme and genes encoding the enzymes methylene-H_4_F dehydrogenase (*mtdA*) and methenyl-H_4_F cyclohydrolase (*fchA*). The genes encoding an NAD-dependent formate dehydrogenase were also present. Genes encoding key enzymes of the serine pathway hydroxypyruvate dehydrogenase (*hpr*) and serine-glyoxalate aminotransferase (*sga*) were present, but the genome of LS7-MT lacked *hps* which encodes hexulose monophosphate synthase, a key enzyme of the ribulose monophosphate pathway. The absence of genes encoding ribulose-1,5 bisphosphate carboxylase/oxygenase (RuBisCO) suggests, therefore, that carbon assimilation in strain LS7-MT is via the serine cycle.

Interestingly, the LS7-MT draft genome contains parts of the methanogenesis pathway for folate biosynthesis. The enzyme formylmethanofuran dehydrogenase subunit C converting formylmethanofuran to CO_2_ and methanofuran was detected. Also, the ffsA gene formylmethanofuran-tetrahydromethanopterin formyltransferase was detected previously reported in *Hyphomicrobium* sp (Strain MC1). This protein catalyzes the transfer of a formyl group from 5-formyl tetrahydromethanopterin (5-formyl-H_4_MPT) to methanofuran (MFR) so as to produce formylmethanofuran (formyl-MFR) and tetrahydromethanopterin (H_4_MPT) (UniProtKB). The formylmethanofuran dehydrogenase enzyme represents the initial reversible step (i.e., found in thermophilic archaea) that produces formylmethanofuran from CO_2_. It is considered that a complex of formyltransferase and formylmethanofuran dehydrogenase converts N5-Formyl H4MPT to formate in many methylotrophic bacteria (as in *Methylobacterium extorquens* AM1) [37].

Genes encoding the MO-Fe-nitrogenase were not detected, indicating that strain LS7-MT is unable to fix N_2_, which confirms our earlier growth studies. Genes encoding glutamine synthetase (*glnA*) and glutamate synthase (*gltBD*) are present, suggesting that ammonia assimilation is via the GS/GOGAT pathway. No genes encoding methylamine dehydrogenase were found but genes encoding the N-methylglutamate pathway (*gmaS*) were present, suggesting that strain LS7-MT assimilates methylamine via this pathway. Trimethylamine is probably metabolized via TMAO since *tmm*, encoding trimethylamine monooxygenase, is present in the genome of strain LS7-MT.

#### Description of *Methylothermalis aethiopiae* Strain LS7-MT gen. nov. sp. nov.

*Methylothermalis* (Me.thy.lo.ther’mal.is) N.L. n. methyl, the methyl group; N.L. pref. methylo, relating to the methyl radical N.L. masc, subst. from Gr. adj. *thermalis* referring to an alkaline thermal spring, and *aethiopiae* (ae.thi.o.pi.ae) referring to the territory where the bacterium was found.

Gram-stain-negative, non-motile, aerobic and small rod-shaped cells that reproduce by binary fision. Cells are of a diameter of 0.3 to 0.5 µm and a length of 0.4–1.5 µm. Very shiny white colonies on gelrite plates were 1.2 to 1.5 mm in diameter. It is a moderate thermophile and facultative methylotroph utilizing methanol and a variety of multi-carbon compounds in addition to sugar via the serine pathway. C18:0, C18:1w7c, C19:0cyc w8c are the dominant cellular fatty acids. It does not grow on methane, and it utilizes nitrate as a nitrogen source. It is not capable of fixing atmospheric nitrogen. Vitamins are required for its growth. It contains MDH, but not pMMO- or sMMO genes. The gene *xoxF1* is present. Optimum growth occurs at 55 °C (range spanning 30 to 60 °C), at pH 6.0 to 9.3 and in the presence of 0.5% (*w*/*v*) NaCl. Phylogenetically, strain LS7-MT belongs to the order *Hyphomicrobiales* of the class *Alphaproteobacteria*. The most closely related extant genera are *Methyloceanibacter* and *Methyloligella* within the family *Hyphomicrobiaceae*. The accession number (in the JGI IMG/ER) of the deposited genome sequences is 2517572012. Genome sequencing revealed a genome size of 2.95 Mbp and a DNA G + C content of 65.9%. The strain LS7-MT was isolated from a terrestrial alkaline hydrothermal spring located in the Ethiopian Rift Valley.

## 4. Conclusions

The novel moderately thermophilic bacterium, strain LS7-MT, was recovered from a methane-utilizing mixed-culture originating from a thermal spring sediment in the Ethiopia Rift Valley. In addition to methanol, strain LS7-MT grew on a range of one-carbon compounds and sugars. Interestingly, it can also grow on a wide selection of sugars, including disaccharides, hexoses and pentoses. Strain LS7-MT is also able to grow on methylamine. Strain LS7-MT is, to our knowledge, the first moderately thermophilic, non-pigmented and facultatively methylotroph, which is affiliated with the order *Rhizobiales* (*Hyphomicrobiales*) of the family *Hyphomicrobiaceae* in the class *Alphaproteobacteria*, to be isolated from a hotspring environment. Based on the initial characterization of the strain LS7-MT, we suggest the name *Methylothermalis aethiopiae* (methyl-, pertaining to the methyl radical; *thermalis* meaning heat; *aethiopiae* meaning “of Ethiopia”) defining its methylotrophic nature, its affiliation with thermophilic environments, and country of origin. The genome of strain LS7-MT will also provide new insights into the diversity of biological methanol oxidation and on the adaptation of this process to thermophilic conditions. Due to its thermophilic nature, this facultative methylotroph might be an interesting candidate for biotechnological applications and further studies on its molecular biology and biochemistry are needed to investigate potential uses in industry.

## Figures and Tables

**Table 1:** Major characteristics of strain LS7-MT and other related alphaproteobacterial strains (thermophiles, moderately thermophiles and mesophiles). Strains: 1, The present study (strain LS7-MT); 2, *Methyloceanibacter caenitepidi* Gela4^T^ [18]; 3, *Methyloligella solikamskensis* SK12^T^ [19]; 4, *Hyphomicrobium* spp. [14]; 5, *Methyloterrigena soli* M48^T^ [33]; 6, *Rubellimicrobium thermophilum* C-Ivk-R2A-2^T^ [21]; 7, *Chelatococcus sambhunathii* HT4^T^ [22]; 8, *Tepidamorphus gemmatus* CB-27A^T^ [23]; 9, *Elioraea thermophila* YIM 72297^T^ [24]; 10, *Porphyrobacter tepidarius* OT3^T^ [25]. Fac., Facultative; +, Positive; −, negative; nr, not reported.

Characteristics	1	2	3	4	5	6	7	8	9	10
Ecology (Habitats)	Alkaline thermal spring	Marine sediment	Saline environment	Soil, water, sewage	Contaminated soil	Paper mill (coloured biofilms)	Hot spring sediment	Hot spring water	Hot spring sediment	Brackish Hot spring
Methylotrophy (M)	Fac. M.	Fac. M.	Obligately M.	Fac. M.	Fac. M.	Strictly aerobic	Fac.	Fac.	Fac.	Fac.
Cell morphology	Rods	Rods			Short rods	Rods	Rods	Rods	Curved rods	Rods
Cell length (µM)	0.4–1.5	2.8–10.4	1.8–2.0	0.5–5.0	1.0–1.5	2.0–4.0	0.5–5.0	0.5–2.0	2.2–3.2	0.5–5.0
Colony colour	Shiny white	White	Colourless	Brownish	Cream	Red-pigmented	Brownish	Non-pigmented	Light pink	Orange
Flagellation	−	−	−	+	+	−	+	−	−	+
Hyphae formation	−	−	−	+	−	−	−	−	−	−
Temp. (opt.) °C	30–60 (55)	19–43 (35)	10–40 (29)	5–45 (28–35)	20–37 (30)	25–55 (45–54)	20–50 (37–42)	30–53 (45–50)	45–60 (55)	30–53 (40–48)
Utilization of:										
methanol	+	+	+	+	+	−	−	−	+ ^a^	−
Methane	−	−	−	−	−	−	−	−	−	−
methylamine	+	+	+	+	nr	+	+	−	nr	+
acetate	+	+	−	+	−	+	+	+	+	+
pyruvate	+	nr	nr	nr	nr	nr	nr	+	+	−
glucose	+	−	−	+/−	+	+	+/−	+	+	+
mxaF gene	+	+	+	+	+	−	−	−	nr	−
Optimum pH	7–8	6–8	7–8	6.5–7.5	7–8	6.5–7.5	7.5–8.0	7.5–8.5	7–7.5	6.5–7.5
NaCl tolerance (%)	0.5	9	16	5.5	0–3.5	5.5	0.0–3.0	0–3	0.5	5.5
G + C content (%)	65.9	63.9	60.5	59–65	60.5	70.0	59–65	66.9	70.8	65.0

**^a^** It has been reported in the paper that *Elioraea thermophila* could grow on methanol, but the *mxaF* or *xoxF* genes were not verified.

**Table 2:** Fatty acid profiles of strain LS7-MT and other related alphaproteobacterial species. Strains: 1, The present study (strain LS7-MT); 2, *Methyloceanibacter caenitepidi* Gela4^T^ [18]; 3, *M. solikamskensis* SK12^T^ [19]; 4, *Hyphomicrobium* spp. [14,35]; 5, *Methyloterrigena soli* M48^T^ [33]; 6, *Rubellimicrobium thermophilum* C-Ivk-R2A-2^T^ [21]; 7. *Chelatococcus sambhunathii* HT4^T^ [22]; 8, *Tepidamorphus gemmatus* CB-27A^T^ [23]; 9, *Elioraea thermophila* YIM 72297^T^ [24]; 10, *Porphyrobacter tepidarius* OT3^T^ [25]. nr, not reported. Values are percentages of total fatty acids. The major fatty acid is shown in bold. Summed features are groups of two or three fatty acids that it cannot be separated by GLC with the MIDI system. Summed feature 7 contains C19:1 ω6c/.846/19cy and Summed feature 8 contains C18:1ω7c and/or C18: 1ω6c.

Fatty Acid	1	2	3	4	5	6	7	8	9	10
C14:0 3-OH	3.99		0.2	2.0–2.8			3.0	1.6		
C16:0 2-OH									5.4	
C16:0 3-OH				1.8–3.4						
**C16:0**	**2.28**	**1.6**	**0.6**	**1.5–2.5**	**23.6**	**22.34**	**1.2**	7.4	**12.6**	nr
C17:0 cyclo						0.48				
C17:0					2.0	0.44	1.5	0.7		
C18:1w9										
C18:1w7c 11-Me			4.8		27.9	0.92		8.3		
**C 18:1w7c**	**24.20**	**60.2**	**36.7**	**74.4–85.2**	**12.1**	4.49	**73.1**	**3.7**	**35.8**	
**C 18:0**	**40.45**	**36.6**	**17.4**	**3.1–7.6**	9.4	**21.62**	**1.7**	**16.2**		
C 18:0 3-OH	4.68				4.5		3.4	2.3	2.5	
C 18:0 2-OH									1.5	
C 18:1 2-OH							7.1		5.6	
**C 19:0 cyc w8c**	**20.77**		**28.7**		**15.3**	**43.91**	**7.8**	**58.2**	**4.2**	
C19:0		1.6		0.1–2.2						
C19:1	0.67									
C 20:2 w6,9c	1.98				0.6	0.66		0.5		
C 20:1 w7c	0.99						0.8	0.7		
C 20:1 w9			5.2							
C 20:0			1.7							
C24:0 3-OH			1.7							
C 25:0 3-OH			1.4							
C 25:0 2-OH										
C 26:0 3-OH			1.6		1.5					
C 10:0 3-OH						1.28				
Summed feature 2						1.2			1.8	
Summed feature 7									**30.1**	
Summed feature 8 Growth (°C)	55	35	29		30	45	30	45	50	

**Table 3:** Genome features of the strain LS7-MT.

Characteristics	Number	% of Total
Genome size (total number of bases)	2,954,375	100
DNA coding number of bases	2,669,286	90.35
DNA G + C number of bases	1,947,002	65.90
DNA scaffolds	237	100
Total number of genes	3152	100
Protein coding genes	3098	98.29
RNA genes	54	1.71
Number of rRNA genes (16S, 23S, and 5S)	3	0.10
Number of tRNA genes	46	1.46
Protein coding genes with function prediction	2355	74.71
Protein coding genes without function prediction	743	23.57
Protein coding genes with enzymes	752	23.86
Protein coding genes connected to Transporter Classification	315	9.99
Genes in Biosynthetic Clusters	94	2.98
Protein coding genes coding signal peptides	367	11.64
Protein coding genes coding transmembrane proteins	834	26.46

## Data Availability

Sequences has been added to the JGI IMG/ER database under the accession number 2517572012, and NCBI, genebank accession number, KP771710 and KP860948.
